# Prevalence of Psychological Symptoms and Its Impact on the Quality of Life of Sickle Cell Disease Patients in Makkah, Saudi Arabia

**DOI:** 10.7759/cureus.32195

**Published:** 2022-12-05

**Authors:** Mohammad Dairi, Shumok S Almatrfi, Manar M Alsharif, Bushra A Fatani, Orjuwan A Almatrafi, Ameerah S Mandourah, Reham M Mashat, Abdulaziz H Basha Ahmed

**Affiliations:** 1 Internal Medicine, Umm Al-Qura University, Makkah, SAU; 2 Medicine, Umm Al-Qura University, Makkah, SAU; 3 Nutrition, King Khalid University, Abha, SAU; 4 Internal Medicine/Hematology, Al Noor Specialist Hospital, Makkah, SAU

**Keywords:** depression, saudi arabia, quality of life, prevalence, adult sickle cell anemia, sickle cell disease (scd)

## Abstract

Background

Patients with sickle cell disease (SCD), which is an inherited autosomal recessive disorder, experience a broad range of symptoms and complications such as acute chest syndrome, infections, strokes, seizures, etc. The main objective of this study was to assess the prevalence of depressive symptoms among SCD patients and determine the impact of sociodemographic factors and genotypes on depressive symptoms in Makkah City, Saudi Arabia.

Methods

A cross-sectional study was conducted among SCD patients aged 18 and above in the outpatient clinics in Makkah’s four main hospitals (Al Noor Specialist Hospital, King Faisal Hospital, King Abdulaziz Hospital, and Hera General Hospital) from December 2021 to April 2022. A total of 222 patients used a self-administrative questionnaire with an Arabic version of Patient Health Questionnaire-9 (PHQ-9) to assess for depression. Data were analyzed using IBM SPSS Statistics, version 26, and the chi-square (χ^2^) test was applied to examine the relationship between the variables. A p-value of less than 0.05 was regarded as statistically significant.

Results

The overall prevalence of depression was 85.6%, and multivariate analysis showed that age between 18 and 30 had a significant statistical value for the prevalence of depression (p-value = 0.029). Univariate analysis also indicated that low levels of education (p-value = 0.037) and low monthly income (p-value = 0.017) had a significant association with depression prevalence.

Conclusion

There is a high prevalence of depression among SCD patients in the Makkah region. Therefore, we recommend establishing a regular regional screening program and psychiatry referral for this patient population.

## Introduction

Sickle cell disease (SCD) is an inherited autosomal recessive disorder caused by a mutation in the β‐globin gene that tends to polymerize the red blood cells into sickle-shaped cells leading to chronic hemolytic anemia [1]. SS hemoglobinopathy (HbSS) is the most frequent and severe form of SCD while SC hemoglobinopathy (HbSC) is usually milder [2]. Patients with SCD experience a broad range of symptoms and complications such as acute chest syndrome, infections, strokes, seizures, priapism, pulmonary hypertension and embolism, deep venous thromboses and retinopathy; many other serious complications can also affect patients’ quality of life, thus increasing morbidity and mortality [3,4].

In Saudi Arabia, numerous studies have been conducted to trace the natural history of SCD. There is a relatively common genetic disorder in this region of the world that is more prevalent in the eastern and southwestern regions [5]. The carrier status for SCD in Saudi Arabia ranges from 2% to 27%, and up to 1.4% have SCD [6]. In this context, the Saudi Ministry of Health introduced the National Premarital Screening Program (NPSP) as a means of prevention of inherited hemoglobinopathies, for all Saudi couples, to reduce the overall disease burden resulting from having affected children [5].

Similar to other chronic medical conditions, SCD is commonly complicated by psychological symptoms that can be attributed to the chronic nature of the disease and other illness-related symptoms [5]. The prevalence of depression in SCD patients has been estimated to be between 21.6% and 44% worldwide and is associated with increased hospitalizations, pain crises, and poor quality of life [3,7]. These findings highlight the significance of detecting and treating psychological symptoms in adults with SCD, especially anxiety and depression.

Previous studies conducted in the southern and eastern regions of Saudi Arabia have found a high prevalence of depressive symptoms of 85.9% and 48.2%, respectively [5,8]. Therefore, in this study, we assessed the prevalence of depression among patients with SCD in four centers in Makkah City in western Saudi Arabia between December 2021 and April 2022, and then studied its relationship with sociodemographic factors.

## Materials and methods

Study design

This was a cross-sectional descriptive study conducted between December 2021 and April 2022 at the outpatient clinics of (Al Noor Specialist Hospital, King Faisal Hospital, King Abdulaziz Hospital, and Hera General Hospital) in Makkah, Saudi Arabia. The required ethical approval for this research was received from the Institutional Review Board (IRB) of the Ministry of Health, Makkah region (IRB no. H-02-K-076-0921-573).

Sample size and inclusion criteria

The targeted sample size was calculated to be 370 using the Raosoft sample size calculator (Raosoft, Inc., Seattle, WA) available online with a margin error determined as 5%, confidence level determined as 95% and an estimated total population of 10,000. The convenience sampling technique was used and a total of 222 patients were enrolled in the study. Patients with adult sickle cell disease, based on hemoglobin electrophoresis, aged 18 years and older were included in the study. Pregnant women, patients with SCD traits, and those who had already been diagnosed with depression were excluded.

Data collection

Data were collected with a self-administered online questionnaire, and patients’ consent was required to start the questionnaire. The questionnaire contained three sections. The first section was general questions regarding patients' sociodemographic data including age, gender, nationality, marital status, level of education, occupation, monthly income, and residential state. The second section assessed sickle cell disease and consisted of nine questions including the age of onset, sickle cell genotype, medication used, number of vaso-occlusive crises (VOCs), hematology clinic visits, and hospitalizations per month. In the last section, the Patient Health Questionnaire-9 (PHQ-9) was used to identify and measure depression severity [9]. A patient's overall score indicated whether they were depressed or not: a score of 0-4 indicated no depression, a score of 5-9 indicated mild depression, a score of 10-14 indicated moderate depression, a score of 15-19 indicated moderate-severe depression, and a score of 20-27 indicated severe depression.

Statistical analysis

Data were analyzed using IBM SPSS Statistics, version 26 (IBM Corp., Armonk, NY). The chi-square (χ^2^) test was used for qualitative data and expressed as numbers and percentages to examine the relationship between the variables. The odds ratio was calculated at a confidence interval (CI) of 95% to assess the risk factors (independent predictors) of depression. A p-value of less than 0.05 was considered statistically significant.

## Results

This study included 222 participants and the majority (63.5%) were in the age range of 18-30 years. Of these, 55% were females, and 92.3% were Saudi nationals; 65.3% of them were single, 49.5% had a bachelor's degree, 27.9% were employed, and 26.6% had a monthly income <5000 Saudi Riyal, or SR (Table 1).

**Table 1:** Distribution of patients according to their demographic data (N=222) SR, Saudi Riyal

Variable	No. (%)
Age (years)	
18-30	141 (63.5)
31-50	81 (36.5)
Gender	
Female	122 (55)
Male	100 (45)
Nationality	
Saudi	205 (92.3)
Non-Saudi	17 (7.7)
Marital status	
Married	70 (31.5)
Single	145 (65.3)
Widow	1 (0.5)
Divorced	6 (2.7)
Educational level	
Primary education	8 (3.6)
Intermediate education	9 (4.1)
Secondary education	79 (35.6)
Bachelor	110 (49.5)
Postgraduate education	10 (4.5)
None	6 (2.7)
Occupation	
Student	58 (26.1)
Employee	62 (27.9)
Other	102 (45.9)
Monthly income (SR)	
<5000	59 (26.6)
5000-10,000	31 (14)
10,000-15,000	18 (8.1)
>15,000	6 (2.7)
None	108 (48.6)

A majority (54.5%) of patients had HbSC, and 42.8% of them had frequent VOC per month. Regarding medical care, 26.1% and 22.5% had frequent visits to the hematology clinic and frequent hospitalizations, respectively. Most patients (81%) attempted to manage their crises at home, and the most common reason for doing so (44.65%) was that they did not believe medical assistance was needed (Table 2).

**Table 2:** Distribution of patients according to their clinical data (N=222) SCD, sickle cell disease; G6PD, glucose-6-phosphate dehydrogenase; VOC, vaso-occlusive crisis; ER, emergency room

Variable	No. (%)
Which type of SCD have you been diagnosed with?	
SS disease	55 (24.8)
SC disease	121 (54.5)
Sß0 thalassemia (beta zero)	13 (5.9)
Sß+ thalassemia (beta plus)	14 (6.3)
Other (thalassemia carrier, G6PD deficiency)	19 (8.6)
Number of VOC per month	
Rare	88 (39.6)
Frequent	95 (42.8)
Other	39 (17.6)
How often do you visit the hematology clinic per month?	
Rare	132 (59.5)
Frequent	58 (26.1)
Other	32 (14.4)
How often you needed to be hospitalized	
Rare	103(46.4)
Frequent	50 (22.5)
Other	69 (31.1)
Have you ever tried to manage pain crisis at home?	
Yes	182 (82)
No	40 (18)
If yes, why did you choose to manage a severe pain crisis at home?	
An unpleasant experience in the emergency room or hospital	15 (11.5)
No transportation	14 (10.8)
No health insurance	3 (2.3)
There is no need for medical assistance	58 (44.6)
Getting medical assistance does not help manage the pain	5 (3.8)
Medical practitioners do not fully understand the nature of the disease or how to treat it	7 (5.4)
It's expensive to go to the hospital every time I'm in pain	3 (2.3)
Nearby hospitals and ERs are not available	8 (6.2)
Other	2 (1.5)
None	15 (11.5)

Almost half of the participants (49.8%) reported that it was somewhat difficult to work, take care of things at home, and/or interact with others due to these problems. Of these, 80.6% received support from family or friends after being diagnosed with SCD, and 31.2% reported that they often needed assistance with daily activities. Housework (27.7%) and transportation (18.1%) were the most common forms of regular support patients needed from their caregivers. Many participants (47.1%) believed that their monthly income would be higher if they did not suffer from SCD (Table 3).

**Table 3:** Distribution of patients according to disease burden, support, and effect on monthly income (N=222) SCD, sickle cell disease

Variable	No. (%)
How difficult has it been for you to take care of your work, take care of things at home, and interact with others due to these problems?	
Not difficult at all	75 (33.9)
Somewhat difficult	110 (49.8)
Very difficult	22 (10)
Extremely difficult	14 (6.3)
Did you receive support from family or friends when you were diagnosed with SCD?	
No	42 (18.9)
Yes	179 (80.6)
How often do you need someone to help you with your daily activities?	
Never	22 (10)
Rarely	21 (9.5)
Sometimes	109 (49.3)
Often	69 (31.2)
What kind of regular support do you need from your caregiver as a result of your SCD?	
Transportation	17 (18.1)
Housework	26 (27.7)
Personal care assistance	8 (8.5)
Healthcare assistance	10 (7.6)
Managing finances	5 (5.3)
Help plan and organize everyday activities	2 (2.1)
Help with childcare	2 (2.1)
Help with schoolwork/tutoring	8 (8.5)
Other	7 (7.4)
How would your monthly income be different if you did not have SCD?	
Monthly income would be higher	104 (47.1)
Monthly income would be lower	8 (3.6)
No change in monthly income	62 (28.1)
Prefer not to say	47 (21.3)

**Figure 1:**
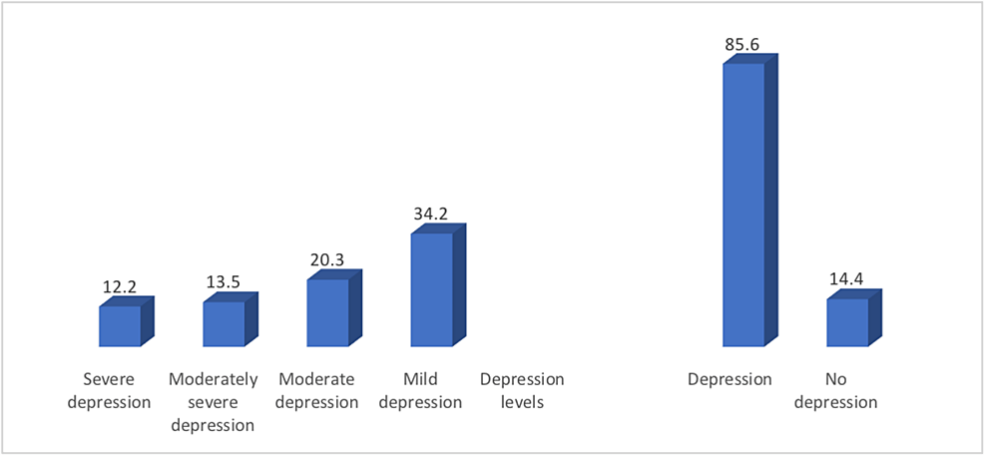
Percentage distribution of patients according to depression prevalence and its levels (N=222)

The prevalence of depression among studied patients was 85.6%. We found that 34.2% had mild depression, 20.3% had moderate, 13.5% had moderately severe, and 12.2% had severe depression (Figure 1).

**Figure 2:**
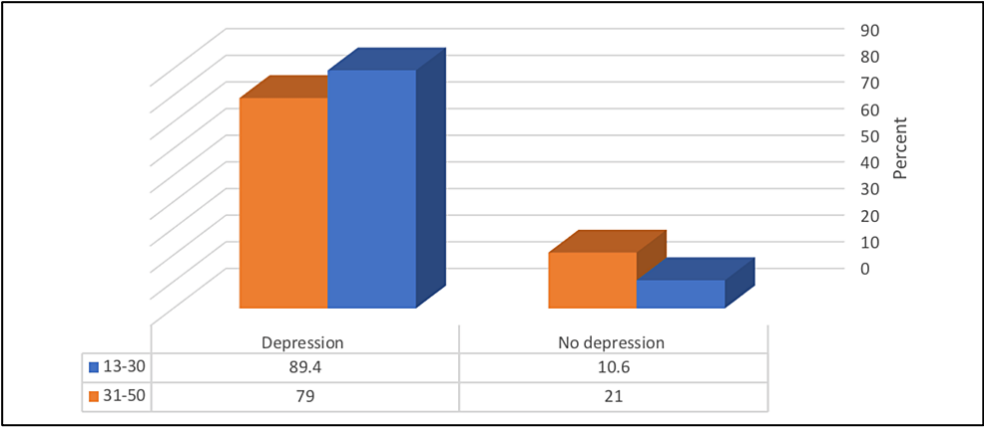
Relationship between depression prevalence and patients' age χ^2^ = 4.46, p-value = 0.035

Younger patients (18-30 years) had a significantly higher prevalence of depression compared with older patients (89.4% vs. 79%) (p≤0.05) (Figure 2).

At the same time, patients who had a lower level of education (intermediate education) and a monthly income of 5000-10,000 SR had a significantly higher prevalence of depression (p≤0.05) (Table 4).

**Table 4:** Relationship between depression prevalence and demographic characteristics of patients (N=222) SR, Saudi Riyal

Variable	Depression		χ^2^	p-value
	No	Yes		
	No. (%)	No. (%)		
Gender				
Female	15 (12.3)	107 (87.7)	0.98	0.321
Male	17 (17)	83 (83)		
Nationality				
Saudi	31 (15.1)	174 (84.9)	1.08	0.297
Non-Saudi	1 (5.9)	16 (94.1)		
Marital status				
Married	14 (20)	56 (80)	3.42	0.331
Single	18 (12.4)	127 (87.6)		
Widow	0 (0.0)	1 (100)		
Divorced	0 (0.0)	6 (100)		
Educational level				
Primary education	4 (50)	4 (50)	11.86	0.037
Intermediate education	1 (11.1)	8 (88.9)		
Secondary education	7 (8.9)	72 (91.1)		
Bachelor	18 (16.4)	92 (83.6)		
Postgraduate education	2 (20)	8 (80)		
None	0 (0.0)	6 (100)		
Occupation				
Student	6 (10.3)	52 (89.7)	1.34	0.511
Employee	11 (17.7)	51 (82.3)		
Other	15 (14.7)	87 (85.3)		
Monthly income (SR)				
<5000	8 (13.6)	51 (86.4)	12.03	0.017
5000-10,000	3 (9.7)	28 (90.3)		
10,000-15,000	7 (38.9)	11 (61.1)		
>15,000	2 (33.3)	4 (66.7)		
None	12 (11.1)	96 (88.9)		

On the other hand, a non-significant relationship was found between depression prevalence and patients' clinical data, disease burden, support, and patients' opinion about the effect of the disease on their monthly income (p≥0.05) (Tables 5, 6).

**Table 5:** Relationship between depression prevalence and patients' clinical data (N=222) SCD, sickle cell disease; G6PD, glucose-6-phosphate dehydrogenase; VOC, vaso-occlusive crisis; ER, emergency room

Variable	Depression		χ^2^	p-value
	No	Yes		
	No. (%)	No. (%)		
Which type of SCD have you been diagnosed with?				
SS disease	11 (20)	44 (80)	3.74	0.441
SC disease	16 (13.2)	105 (86.8)		
Sß0 thalassemia (beta zero)	0 (0.0)	13 (100)		
Sß+ thalassemia (beta plus)	2 (14.3)	12 (85.7)		
Other (thalassemia carrier, G6PD deficiency)	3 (15.8)	16 (84.2)		
Number of VOC per month				
Rare	14 (15.9)	74 (84.1)	1.17	0.556
Frequent	11 (11.6)	84 (88.4)		
Other	7 (17.9)	32 (82.1)		
How often do you visit the hematology clinic per month?				
Rare	20 (15.2)	112 (84.8)	0.35	0.837
Frequent	7 (12.1)	51 (87.9)		
Other	5 (15.6)	27 (84.4)		
How often you needed to be hospitalized?				
Rare	16 (15.5)	87 (84.5)	2.26	0.322
Frequent	4 (8)	46 (92)		
Other	12 (17.4)	57 (82.6)		
Have you ever tried to manage pain crisis at home?				
Yes	26 (14.3)	156 (85.7)	0.01	0.907
No	6 (15)	34 (85)		
If yes, why did you choose to manage a severe pain crisis at home?				
An unpleasant experience in the emergency room or hospital	1 (6.7)	14 (93.3)	13.63	0.136
No transportation	1 (7.1)	13 (92.9)		
No health insurance	0 (0.0)	3 (100)		
There is no need for medical assistance	17 (29.3)	41 (70.7)		
Getting medical assistance does not help manage the pain	0 (0.0)	5 (100)		
Medical practitioners do not fully understand the nature of the disease or how to treat it	0 (0.0)	7 (100)		
It's expensive to go to the hospital every time I'm in pain	1 (33.3)	2 (66.7)		
Nearby hospitals and ERs are not available	0 (0.0)	8 (100)		
Other	0 (0.0)	2 (100)		
None	3 (20)	12 (80)		

**Table 6:** Relationship among depression prevalence, disease burden, and monthly income (N=222) SCD, sickle cell disease

Variable	Depression		χ^2^	p-value
	No	Yes		
	No. (%)	No. (%)		
How difficult has it been for you to take care of your work, take care of things at home, and interact with others due to these problems?				
Not difficult at all	15 (20)	60 (80)	3.87	0.276
Somewhat difficult	14 (12.7)	96 (87.3)		
Very difficult	1 (4.5)	21 (95.5)		
Extremely difficult	2 (14.3)	12 (85.7)		
Did you receive support from your family or friends when you were diagnosed with SCD?				
No	10 (23.8)	32 (76.2)	3.64	0.056
Yes	22 (12.3)	157 (87.7)		
How often do you need someone to help you with your daily activities?				
Never	2 (9.1)	20 (90.9)	0.63	0.887
Rarely	3 (14.3)	18 (85.7)		
Sometimes	16 (14.7)	93 (85.3)		
Often	11 (15.9)	58 (84.1)		
What kind of regular help or support do you need from your caregiver as a result of your SCD?				
Transportation	4 (23.5)	13 (76.5)	8.63	0.734
Housework	3 (11.5)	23 (88.5)		
Personal care assistance	2 (25)	6 (75)		
Healthcare assistance	4 (40)	6 (60)		
Managing finances	1 (20)	4 (80)		
Help plan and organize everyday activities	1 (50)	1 (50)		
Help with childcare	0 (0.0)	2 (100)		
Help with schoolwork/tutoring	1 (12.5)	7 (87.5)		
Other	2 (28.6)	5 (71.4)		
How would your monthly income be different if you did not have SCD?				
Monthly income would be higher	10 (9.6)	94 (90.4)	6.18	0.103
Monthly income would be lower	3 (37.5)	5 (62.5)		
No change in monthly income	11 (17.7)	51 (82.3)		
Prefer not to say	8 (17)	39 (83)		

Multivariate logistic regression analysis was done to determine the risk factors (independent predictors) of depression among studied participants. The age of 18-30 years was an independent predictor (risk factor) of depression. While other variables were significant in the univariate analysis, they did not show significance after the multivariate analysis (Table 7).

**Table 7:** Multivariate logistic regression analysis of risk factors of depression among the participants SCD, sickle cell disease; SR, Saudi Riyal

Variable	B	Wald statistic	p-value	Odds ratio (95% CI)
Age	0.89	4.75	0.029	0.4 (0.18-0.91)
Educational level	0.26	1.48	0.224	1.3 (0.85-2)
Monthly income (SR)	0.01	0.008	0.927	1.01 (0.8-1.271)
Which type of SCD have you been diagnosed?	0.14	0.65	0.419	1.16 (0.8-1.66)

## Discussion

Sickle cell disease is one of the most common diseases that cause chronic pain. It can also lead to a change in behavior and depressive symptoms [5]. Here, we assessed the prevalence of depression among SCD patients and determined the impact of sociodemographic factors and genotype on depressive symptoms in Makkah.

In this study, most participants (85.6%) were found to have depression. Our finding is consistent with studies by Alsubaie et al. and Aljumah et al. with depression rates of 85.9% in the southeastern Saudi Arabia and 61.1% in eastern Saudi Arabia, respectively [8,10]. The regional distribution of SCD was more prevalent in the eastern region with a prevalence of 145 per 10,000 followed by the southern region with a prevalence of 24 per 10,000, western region at 12 per 10,000, and central region at 6 per 10,000 [11]. Several factors could have contributed to these results, and these were evaluated as a secondary objective. First, younger patients (aged 18-30 years) had a higher depression prevalence than older patients (aged 30-51 years). The previous literature, however, did not show that age played a significant role. However, Adzika et al. reported significantly higher depression scores among 40- to 49-year-olds than any other age group [12]. In our study sample, 63.5% of study participants were aged 18-30 years, while 36.5% were 31-50 years of age, which may account for this difference. Second, depression is more prevalent among patients with lower levels of education, which is consistent with the study by Schaeffer et al. in which depressed SCD patients were found to be threefold more likely to have less than a secondary level of education [13]. This could be related to their recurrent pain crisis, which results in poor academic performance and missed school days. Third, patients with low incomes are more likely to be depressed. This concurs with the previous literature as Aljumah et al. reported low incomes were significantly associated with depression, at a p-value of 0.019 [14].

Also, as a secondary objective of the study, we determined the prevalence of depressive symptoms among various SCD genotypes. In light of the fact that HbSS is a more severe genotype of SCD, it is assumed to confer a much higher risk of psychological problems than HbSC, which is a less severe genotype. Here, a total of 55 (80%) of HbSS patients and 121 (86%) of HbSC patients were found to have depressive symptoms, thus suggesting that higher psychological distress levels are also associated with milder genotypes of SCD, that is, the HbSC. This result is inconsistent with that of Anim et al. who demonstrated that HbSS patients exceeded HbSC patients in the number and severity of their psychological symptoms [2].

This study supports other previous studies’ findings that disease severity is not necessarily correlated with psychological distress [7,13,15]. Despite worsening health-related quality of life in two domains (general health and role-physical), more severe genotypes did not impact mental health [10]. Our study also showed that patients experiencing frequent VOCs and frequent hospitalizations and clinic visits had a higher risk of depression. These results support the findings of prior studies [3,5,7]. These results are reasonable because a VOC is the major cause of painful events with significant morbidities affecting the course of the disease. Furthermore, most patients (81%) attempted to manage their crises at home, mainly because they believed that medical assistance was not necessary (44.6%). The same belief was also expressed in previous studies by patients (76.6%) in their ability to cope with crises at home [16]. There are a number of potential explanations for these results including inadequate patient education regarding VOC management as well as depression leading to social withdrawal and isolation.

Additionally, while studying the effect of SCD on patients’ daily lives, we found that 49.8% of the participants reported that taking care of work, home, and interacting with others was somewhat difficult. We also found that 27.7% of the participants regularly needed help with housework and 18.1% regularly needed help with transportation. These findings are consistent with Osunkwo et al.’s study in which 2145 SCD patients were surveyed, and 38% reported that SCD affected their ability to do household tasks and 36% reported that it affected their social life [17].

We found that nearly half of the participants (47.1%) believed their monthly income would be higher if they did not have SCD, which is also consistent with the survey by Osunkwo et al. where half of the patients (54%) reported the same [17]. People with SCD often miss work likely due to frequent and unpredictable pain crises and fatigue, which interfere with their ability to work.

A research showed that the number and severity of VOCs negatively impacted work performance, particularly for patients who had four or more crises in the previous year and reported a lack of attendance at work [16]. However, we did not find any statistically significant difference for SCD impacting the monthly income of patients.

This study does have some limitations. First, it is a descriptive study, and thus causal inferences cannot be made. Second, we used a convenient sample from four centers in Makkah, which might be too small to explore factors associated with depressive symptoms among patients with SCD. Third, self-administered surveys may introduce recall and social desirability bias. Fourth, while the PHQ-9 is a common and validated screening tool, a structured clinical interview would have yielded an accurate diagnosis of depression.

## Conclusions

We conclude that there was a high prevalence of depressive symptoms among SCD patients in Makkah, Saudi Arabia. The most important risk factors for depression in SCD patients are young age, high frequency of VOCs, poor educational background, and low monthly income. Future studies on the current problem should focus on utilizing regular screening models, early psychiatric referrals, and follow-up visits for depression. The community should consider psycho-behavioral and/or pharmacological interventions to mitigate the consequences among SCD patients.
